# 2,4-Dichlorophenoxyacetic Acid in the Gas and Crystal Phases and Its Intercalation in Montmorillonite—An Experimental and Theoretical Study

**DOI:** 10.3390/molecules30020367

**Published:** 2025-01-17

**Authors:** Claro Ignacio Sainz-Díaz, Nelly L. Jorge, Jorge M. Romero, André Grand, Alfonso Hernández-Laguna

**Affiliations:** 1 Instituto Andaluz de Ciencias de la Tierra (IACT-CSIC), Consejo Superior de Investigaciones Científicas, Av. de las Palmeras 4, 18100 Armilla, Granada, Spain; 2 Laboratorio de Investigaciones en Tecnología del Medio Ambiente, Área de Química Física, Facultad de Ciencias Exactas y Naturales y Agrimensura, Universidad del Nordeste, Corrientes 3400, Argentina; lidianj@exa.unne.edu.ar (N.L.J.); ing.jorgemromero@gmail.com (J.M.R.); 3 Université Grenoble Alpes, Commissariat à l’Energie Atomique, Centre National de la Recherche Scientifique, Institute for Nanoscience and Cryogenics, Systèmes Moléculaires et NanoMatériaux pour l’Énergie et la Santé, F-38000 Grenoble, France; andre.grand8@wanadoo.fr

**Keywords:** 2,4-dichlorophenoxyacetic acid, DFT, intercalation in clay minerals, dimer, IR assignment, NMR, crystal structure, hydrogen bonds

## Abstract

Many properties of 2,4-dichlorophenoxyacetic acid (2,4-D) depend on its molecular environment, such as whether it is an isolated molecule, a dimer, or in a crystalline state. The molecular geometry, conformational analysis, and vibrational spectrum of 2,4-D were theoretically calculated using Density Functional Theory (DFT) methods. A new slightly more stable conformer was found, which is different to those previously reported. The most stable conformer shows a dimer by means of hydrogen bonds between the carboxylic groups of both molecules, which agrees with the experimental results. The crystal structure of 2,4-D was also calculated with 3D periodical boundary conditions at the DFT level. From the theoretical IR spectra, a vibrational analysis of this molecular species was accomplished, and the bands were reassigned. ^1^H and ^13^C NMR in the dissolution and solid states, respectively, showed intramolecular hydrogen bonds between carboxylic acid groups. The dimer is more stable than the isolated molecule. All these results indicated that the dimer can also exist in the solid state, which could explain the low solubility of this compound. In addition, the intercalation of 2,4-D into the confined interlayer space of montmorillonite was also calculated, and it was found that the adsorption is energetically favourable. This result was experimentally confirmed. These findings predicted that these natural clay minerals, which are found in the environment, can be excellent adsorbents for the 2,4-D pollutant.

## 1. Introduction

Aiming to augment productivity, intensive agricultural exploitations strongly increase the application of fertilizers and pesticides (herbicides, fungicides, insecticides, and others). Most of these chemicals can infiltrate and leach through water, soil profile, and strata subsoil, contaminating soils, surficial water, and groundwater [1,2]. Herbicides derived from phenoxy-acetic acids have important chemical effects and non-desirable pharmacodynamics and pharmacokinetic effects on mammalian species [3,4,5,6]. Phenoxy-acid derivatives are systemic herbicides, which are absorbed by the leaves and/or the roots, that are transported by the sap to the whole body of the plants; these reach the internal tissues, which finally kill the plants. This type of herbicide is considered to be one of the first selective herbicides because it kills many broadleaved plants while causing little damage to the narrow leaves. These derivatives and their metabolites are the most important pollutants of drinking water as they have a very low maximum concentration limit for drinking water. These compounds enter into the formulation of many pesticide preparations and are hazardous to human beings.

The herbicidal activity (HA) of phenoxy-acetic acid is greatly influenced by the substituents on the aromatic group. When a halogen atom—either fluorine or chlorine—is substituted in the aromatic moiety, the HA generally increases. The C-Cl bond is especially stabilized by conjugation with the π electron cloud of the aromatic phenyl group. The position of this halogen also plays a crucial role in relation to the HA [7]. When a hydrogen atom in an aromatic group is substituted by an aliphatic group, the HA does not significantly increase [8]. To increase the activity, the molecule must possess one -COOH group or a group for reacting with plant tissues [8,9]. Therefore, the type and position of substituents are an important issue when looking for a high HA. In this way, a theoretical search about the structural properties of phenoxy acetic compounds would be very useful as a means to start to understand the HA and the toxic properties at the molecular level.

**Figure 1:**
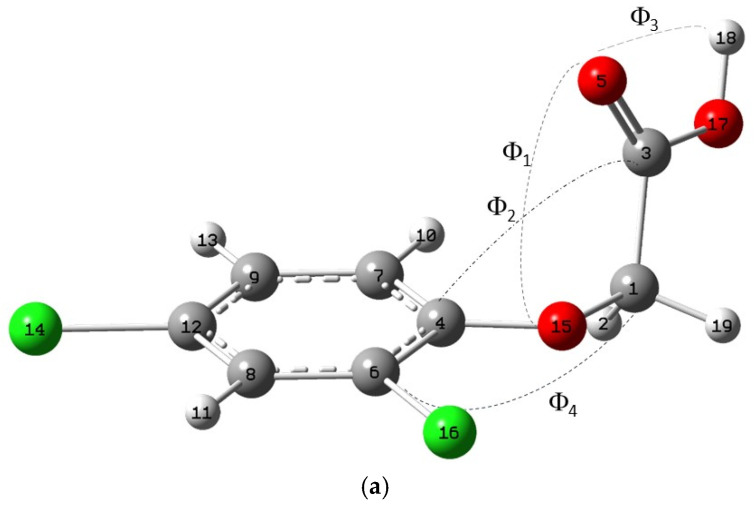

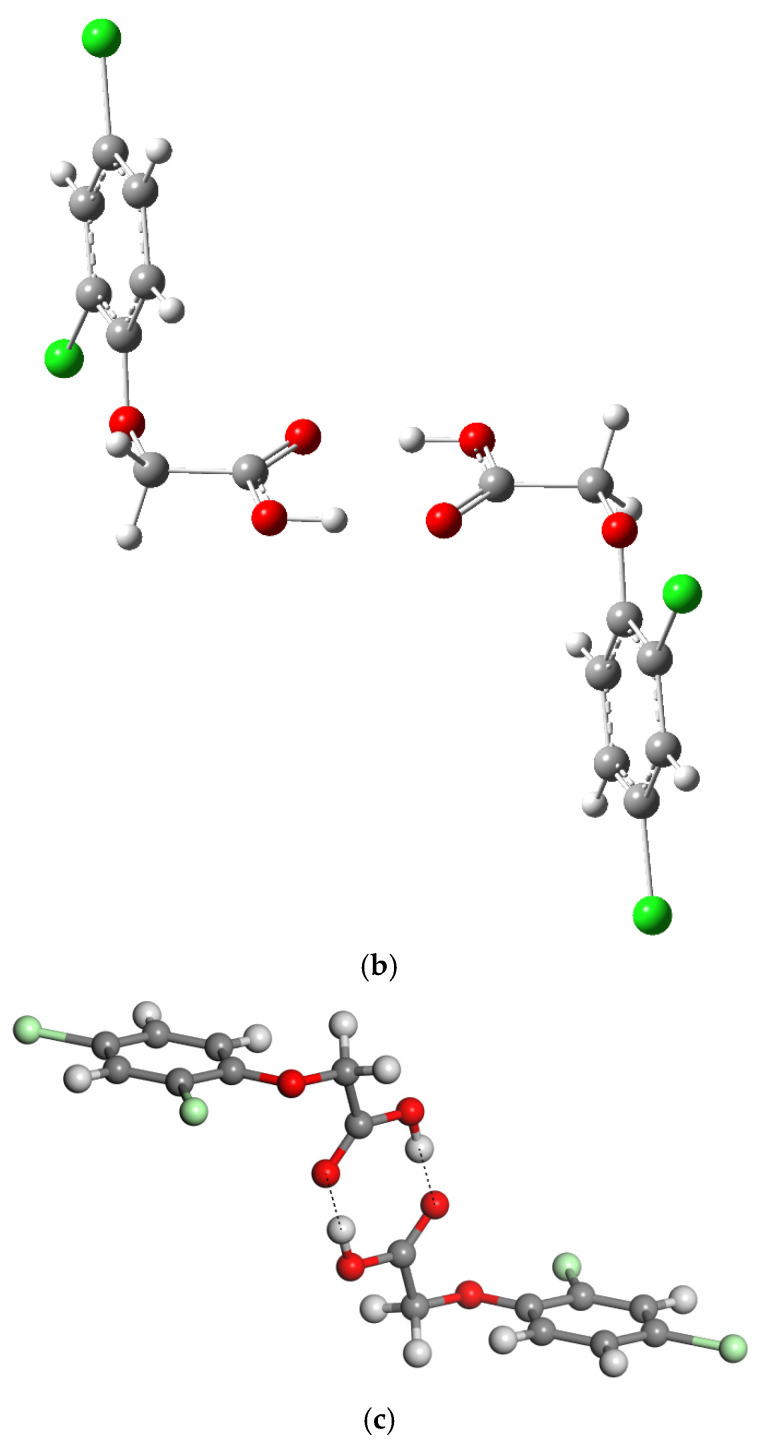
Optimized molecular structure of the most stable conformer I (**a**). Dimer-I of 2,4-dichlorophenoxyacetic acid optimized with B3LYP/6-311++G(d,p) (**b**) and with CASTEP (**c**). The C, H, O, and Cl atoms are in grey, white, red, and green colours, respectively. Dashed lines mean hydrogen bond. This criterium is extended for the rest of this work.

The herbicide 2,4-dichlorophenoxyacetic acid (2,4-D) (Figure 1) widely fulfils the previous HA requirements. Therefore, 2,4-D is a very popular plant growth regulator and is applied as an herbicide for general and broadleaved weed control, especially in the rice ecosystem. This compound has a maximum concentration limit in drinking water of 0.7 μg/L [10], and it is found with a maximum concentration of 0.012 μg/L in underground water sources [11,12] and 1.22 μg/L in river water [11,13] in European Union countries.

In a previous article, the hydrolysis reaction of the 2,4-D was studied [14] by the kinetics of the disappearance of the 2,4-D at particular values of pH and temperature, and a mechanism of the hydrolysis reaction was proposed with a computational model at the gas phase. Theoretical vibrational frequencies were compared with experimental frequencies [15,16,17]. Karthikeyan and Saravanan [18] determined the Raman spectra and calculated the phenoxy-acetic acid and other Cl-derivatives at the Hartree–Fock level, finding the chlorine substitution effect at the molecule. Badawi [16] calculated eight conformers, where the most stables are energetically very close. In this work, we found one new slightly more stable but different conformer with respect to Badawi’s conformers [16]. This molecule can be either an isolated molecule or dimer, due to the strong interactions between the carboxylic groups. Molinelli et al. [19] found that the dimerization rate of 2,4-D is one order of magnitude higher than the interaction with polymers. Previous studies on the crystal structure of 2,4-D [20] by X-ray diffraction experiments showed that, in the solid phase, two molecules are linked together by hydrogen bonds between their carboxylic groups, producing a centre-symmetrical cyclic structure dimer. However, it not well known how the 2,4-D molecules are distributed in the atmospheric aerosols and in the soils and waste waters. A higher knowledge of the molecular interactions of 2,4-D is interesting to investigate the interaction with other soil components like mineral surfaces. Moreover, the molecular environment of this pollutant is critical for the risk evaluation of toxicity and carcinogenicity tests [21] in the interactions with receptors at the molecular level. The use of low-cost adsorbents, like clay minerals, is becoming recognized as a successful method of prevention and remediation environmental strategies. Aquino et al. studied the adsorption of 2,4-D on the Goethite surface [22]. Clay minerals were reported to display excellent adsorbing properties with low prices and easy access to ubiquitous deposits [23].

In this work, the acidic form of the molecule, one dimer in the gas phase, and the crystal structure of 2,4-D were studied at the DFT level, and the vibrational spectrum was calculated, studying the dimer effect on the molecular structure and frequencies. These calculations helped the experimental vibrational spectrum to be assigned. Moreover, the ^1^H and ^13^C NMR spectra of the compound in the dissolution and solid states, respectively, were also recorded in order to understand the intermolecular situation of 2,4-D. In addition, the intercalation of 2,4-D into the interlayer space of montmorillonite was calculated and determined as an energetically favourable process. This study was compared with additional experimental works of the adsorption and desorption of 2,4-D on Argentinian clay-rich soils.

This paper is organized as follows: (i) the Results and Discussion section starts with geometry optimization and a conformational analysis for the molecule, dimer, and crystal structures of 2,4-D; (ii) next, the intercalation of 2,4-D into montmorillonite is examined; (iii) this process is further studied experimentally by exploring the adsorption of 2,4-D on clay-rich soils; (iv) a complete and comparative analysis of the vibrational spectra between the isolated molecule, dimer, and crystal structures is accomplished along with frequency assignments; (v) another section focuses on the NMR methods; and (vi) the work ends with some concluding remarks.

## 2. Results and Discussion

### 2.1. Structure Calculations

#### 2.1.1. Molecule

A conformational analysis was performed for a comprehensive exploration of Potential Energy Surface (PES). Combining several dihedral angles (Figure 1a), nine conformers of the 2,4-D acid molecule were found. All of them were fully optimized and did not show any imaginary frequency. Our results were similar to previous work [16]. However, we found a new conformer that is the most stable (conformer I). In our conformational analysis, the second more stable conformer was II, which is similar to previous reports [16]. The energy differences between both conformers are very low, and both can exist at room temperature. However, an important torsional difference can be observed: the torsional angle ϕ_4_ is 180° in our minimal conformer I corresponding to a *trans* conformation, whereas this angle is close to 90° in conformer II (Table S1). The calculated geometry of conformer I is in accordance with the X-ray experimental data provided by Smith et al. [20]. The herbicide exists predominantly in non-planar conformations at room temperature. The alkylic chain is perpendicular to the aromatic ring plane, the carbonyl bond is in the same plane as the alkoxy O atom, and the hydroxyl bond is coplanar to the carbonyl group. Our full optimized conformer I (Figure 1a) yielded good results, as can be seen from the RMS values of the differences between the experimental and calculated geometrical parameters (Table S1): the smallest RMS values are between the experimental structure and conformer I, especially the RMS of bond lengths and bond angles. Nevertheless, the RMS of the dihedral angles increases to 5.5°. The origin of this discrepancy is likely due to the different phases in which the experiment and calculations were performed. Intramolecular interactions and steric effects play a competitive role in the conformational stability in the ground state. Considering the phenyl moiety of the molecule, the conjugation effects should be important for stabilizing planar structures, whereas the steric forces would stabilize non-planar structures. The steric forces should be responsible for the stability of the I.

The dipole interactions are also important for determining the relative stability of conformations, so conformer I shows a high value of μ (4.22 D), indicating larger stabilization in polar solvents, like water, the solvent applied to weeds. The same conformer was obtained with the CASTEP program, which have important methodological and electronic differences with GAUSSIAN09.

#### 2.1.2. Dimer

2,4-D can build cyclic dimers by hydrogen bond interactions of the carboxylic acid groups (Figure 1b,c). When choosing the two most stable conformers of 2,4-D, two possible dimers can be considered, D-I (with conformer I) and D-II (with conformer II) (Figure S1). The optimization of both dimers indicated that D-I is 1.02 kcal/mol more stable than D-II. The dichlorophenyl groups are in a disposition approximately perpendicular to the cyclic hydrogen bond plane of the carboxylic groups. Calculated geometric parameters are compared with the experimental data of the 2,4-D crystal [20] (Table S2). Once again, the RMS values between the experimental structure and the dimer D-I are lower than that of D-II (Table S2). Generally, the calculated bond lengths for the dimer agree with the experimental values of the crystal, though slight differences are observed in a few bond angles and dihedral angles. Again, this is probably due to the difference in phase. Nevertheless, one can be confident with the results of these calculations. Similar structure D-I was obtained with CASTEP.

Table 1 shows the total energy (^&^E) of the molecule and dimer D-I, including the ZPE (^#^E) corrections. In addition, the BSSE (*E) correction can also be observed for the dimer. From these values, the energy of the interaction between both molecules is −15.0 kcal/mol (considering ZPE). The steric forces also play an important role in stabilizing the ground-state structures of non-planar conformers. The nearly null dipole moments of the dimer indicate that the stabilization is also coming from the fitting of the dipolar moments of the molecules at dimer formation.

#### 2.1.3. Crystal Structure

**Figure 2:**
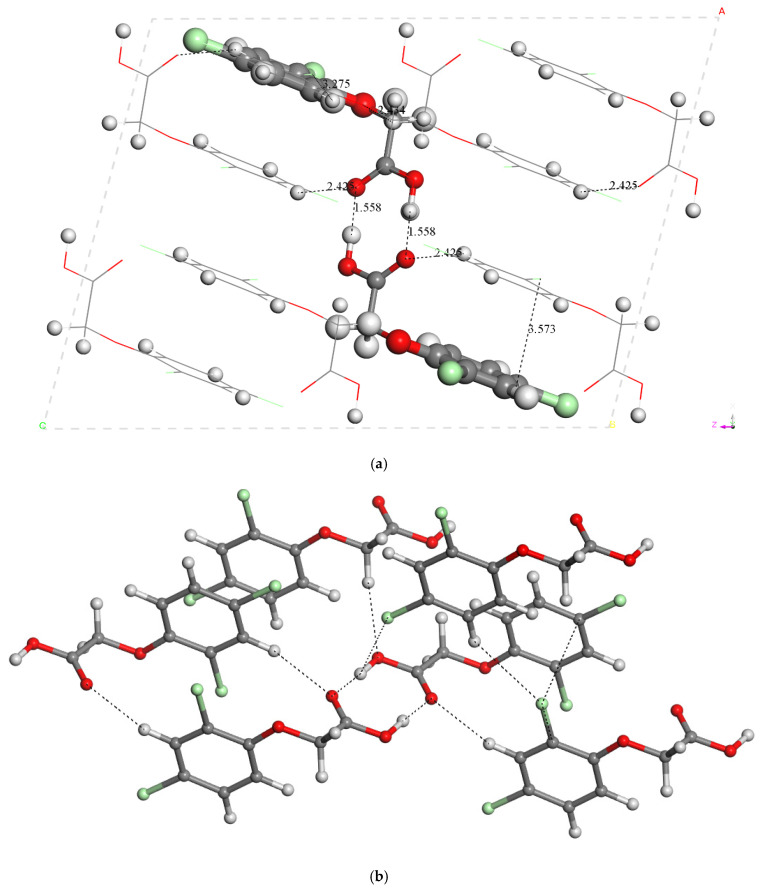

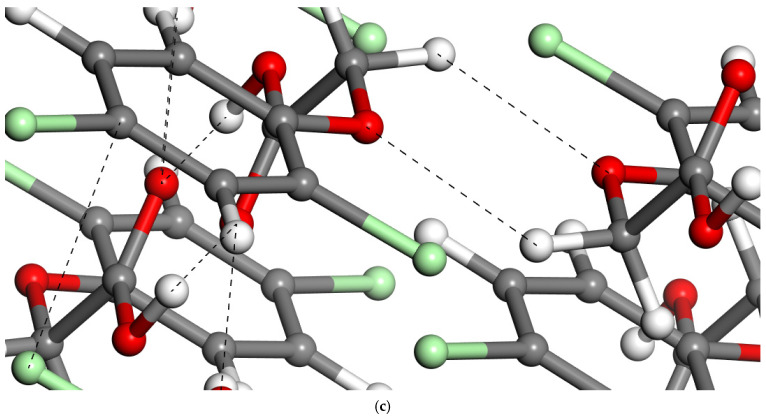
Optimized (CASTEP) crystal structure of 2,4-D showing different intermolecular interactions. View from the (010) plane the dimer structure is highlighted with balls showing a R^2^_2_(8) ring (**a**). A general view (**b**). A zoom view highlighting the R^2^_2_(6) ring (**c**).

The above results get us to examine the crystal structure of 2,4-D. The asymmetric unit of the crystal lattice of 2,4-D was transformed to a P1 symmetry unit cell with two 2,4-D molecules (*Z* = 2) and was calculated with CASTEP and periodical boundary conditions. The crystal cell was fully optimized, including atomic positions and cell parameters, resulting in the following cell parameters: *a* = 7.19 Å, *b* = 7.86 Å, *c* = 8.95 Å, α = 90.6°, β = 104.0°, and γ = 110.7°, which are quite close to the experimental lattice cell parameters of the crystal structure (*a* = 7.12 Å, *b* = 7.84 Å, *c* = 9.0 Å, α = 90.7°, β = 104.6°, γ = 110.5°) (Table 2) [20]. This optimization was performed under different computational conditions, with ultrasoft pseudopotentials at 490 and 630 eV energy cutoffs and with norm-conserving pseudopotentials, and in all cases, the cell parameters were close to the experimental values (Table 3), being the closest to the experimental parameters those calculated with 630 eV and ultrasoft pseudopotentials. The carboxylic groups formed dimers with strong hydrogen bonds, d(CO···HOC) = 1.558 Å. The conformation of the molecule was similar to that found above for the isolated molecule at the gas phase. The conformation of the intra-crystalline dimers was similar that in the gas-phase dimer (Figure 2). This dimerization was not described previously. The carbonyl O atoms and the H-C atoms showed electrostatic interactions, d(C=O···H-C) = 2.425 Å. Intermolecular motifs were found with hydrogen bonds between C-H bonds and carbonyl and ether O atoms, forming alternating R^2^_2_(8) and R^2^_2_(6) rings [24]. 

The cohesion energy *E*_coh_ was calculated in Equation (1) as follows:

*E*_coh_ = *E*_crys_ − 2 *E*_mol_ (1)

where *E*_crys_ is the energy of the optimized crystal structure, and *E*_mol_ is the energy of one 2,4-D molecule optimized in a 3D periodical cubic box with a constant volume of 12 × 12 × 12 Å^3^, at the same computational level of the crystal. The *E*_coh_ was −65.7 kcal/mol.

A dimer of 2,4-D was extracted from this optimized crystal structure, and it was included in a 3D periodical box and optimized. The structure of this dimer is close to the conformation of the above gas phase dimer. The carboxylic groups are joined by both hydrogen bonds with *d*(C=O···HOC) = 1.593 Å, being longer and weaker than in the crystal lattice. The *E* of the hydrogen bonds of this dimer, with respect to the isolated molecules, was −15.8 kcal/mol, which is consistent with the previous values calculated above from isolated molecules. This indicates that the hydrogen bonds between the carboxylic groups is one of the main cohesion forces; however, additional intermolecular interactions contribute to the higher packing energy, that is, *E*_coh_, for instance, electrostatic interactions between Cl and O atoms with H atoms, and all aromatic rings are in a parallel disposition with π-π interactions in an inter-ring distance of 3.579 Å (Figure 2). This interaction is enhanced by the presence of Cl atoms with their π electrons. Moreover, the C-Cl···π, C-H···π, C-H···O, and C-Cl···C interactions participated in the stabilization of packing in the crystal lattice, whereas the aromatic rings were on the opposite side of the isolated dimer.

#### 2.1.4. Intercalation of 2,4-D into Montmorillonite

**Figure 3:**
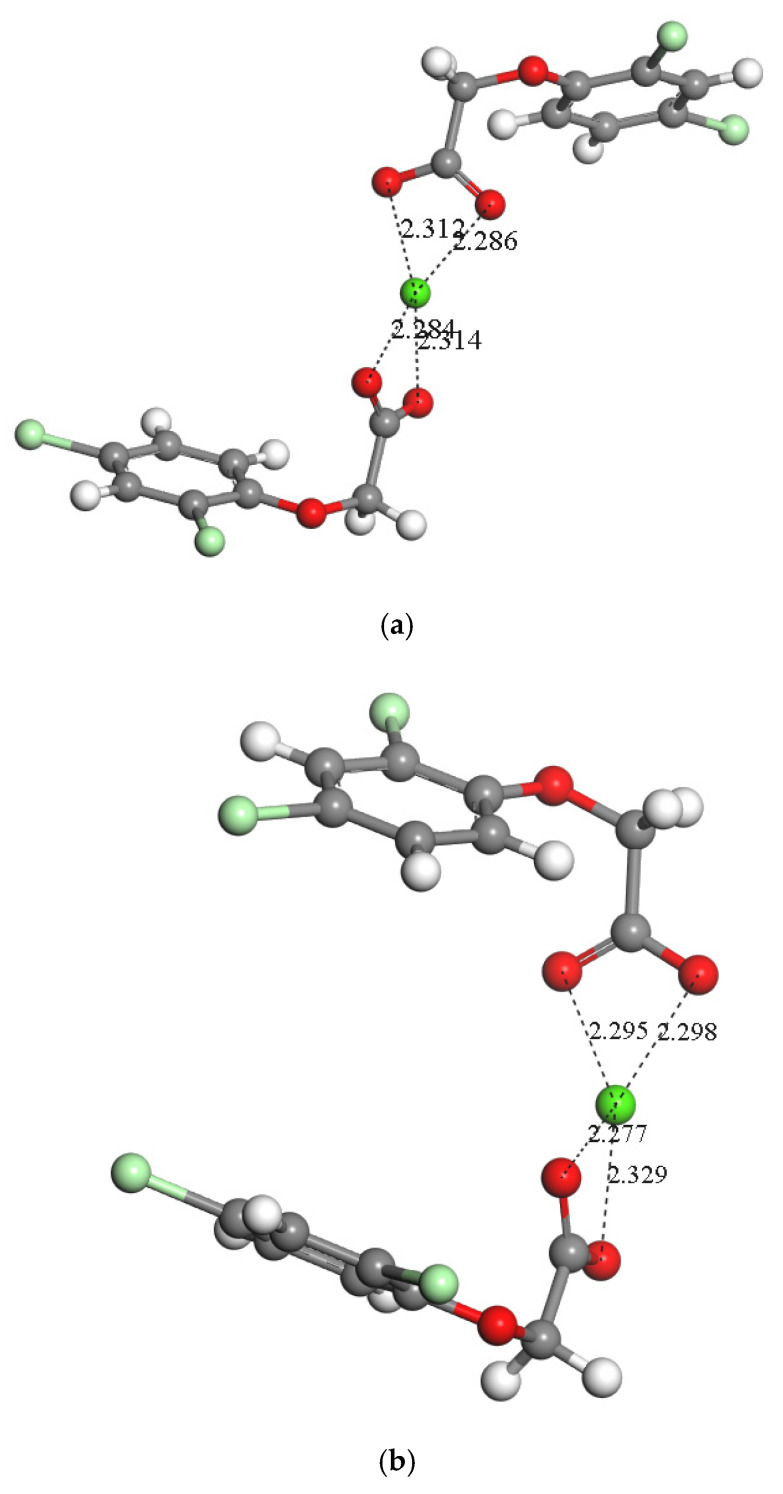

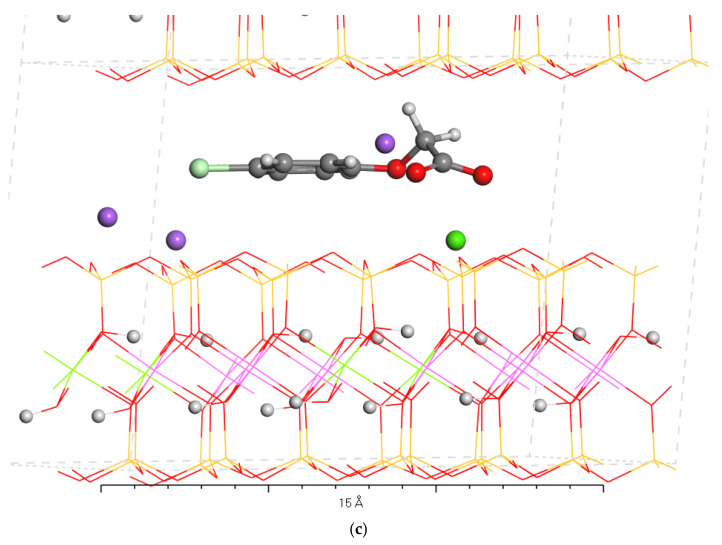
Optimized structure of the (2,4-D)_2_Ca salt, *anti* (**a**) and *syn* (**b**) configuration, and 2,4-D intercalated into montmorillonite (**c**). The H, Si, Al, Mg, Na, Ca, O, C, and Cl atoms are in white, yellow, pink, clear-green, fuchsia, green, red, grey, and pale-green colours, respectively. The H atoms and interlayer atoms are depicted as balls.

An isolated Na salt of 2,4-D was placed in the centre of a 3D periodical box of 20 × 20 × 20 Å^3^ (2,4-DNa) and optimized. The alkoxy chain adopted a perpendicular orientation with respect to the aromatic ring, and the d(CO···Na) distances were 2.22–2.24 Å. Similarly, a Ca salt of 2,4-D was created and optimized, (2,4-D)_2_Ca (Figure 3a,b). Two possible configurations were considered with respect to the aromatic rings: (i) both rings at the opposite side (Figure 3a, *anti*) and (ii) both ring at the same sides (Figure 3b, *syn*). In both complexes, the carboxylic groups coordinated with Ca^2+^ by twisting 90° of each other, and both aromatic rings were not parallel with d(O···Ca) distances of 2.28–2.33 Å (Figure 3). The form *anti* was 0.58 kcal/mol more stable than the *syn* one.

A montmorillonite (MNT) supercell with four interlayer Na^+^ cations (MNTNa_4_) was optimized at a constant volume with the Dmol^3^ method. From this structure, another model of MNT supercell was created with two Na^+^ and one Ca^2+^ cations (MNTNa_2_Ca) and optimized. From MNTNa_4_, a 2,4-DCa^+^ monocation was placed in the centre of the interlayer space of MNT exchanged by one Na^+^ cation (MNTNa_3_Ca-2,4-D) and optimized with Dmol^3^ (Figure 3c). The aromatic ring was parallel to the mineral surface, the (001) plane. The organic molecule was more planar than out of MNT, where the alkoxy group was not perpendicular to the aromatic ring. The carboxylic O atoms are coordinating the Ca^2+^ cation with d(O···Ca) = 2.27–2.35 Å, and one of the carboxylic O atoms is also coordinating one Na^+^ cation with d(O···Na) = 2.29 Å, d(O···Ca) = 2.35 Å. The methylene H atoms are oriented to the basal mineral surface O atoms (Figure 3c).

Then, considering the first and last steps of the intercalation process, the energy of this process can be calculated from Equation (2) as follows:

MNTNa_4_ + (2,4-D)_2_Ca = MNTNa_3_Ca-2,4-D + 2,4-DNa (2)

And the intercalation energy will be calculated from Equation (3) as follows:

*E*_intercal_ = *E*_MNTNa3Ca-2,4-D_ + *E*_2,4-DNa_ − *E*_MNTNa4_ − *E*_(2,4-D)2Ca_ (3)

The intercalation energy of 2,4-D in MNT is −21.87 kcal/mol, similar to the adsorption energy of 2,4-D on the Fe oxide surface reported previously [22].

Depending on the experimental conditions, this process can also be considered by calculating Equation (4) as follows:

MNTNa_2_Ca + 2,4-DNa = MNTNa_3_Ca-2,4-D (4)

And the intercalation energy will be calculated from Equation (4) as follows:

*E*_intercal_ = *E*_MNTNa3Ca-2,4-D_ − *E*_2,4-DNa_ − *E*_MNTNa2Ca_ (5)

The intercalation energy for this process is −93.34 kcal/mol. Both processes indicate that the intercalation of 2,4-D in MNT is energetically favourable.

Applying the COSMO continuum solvation model to simulate an aqueous effect, the intercalation energy in an aqueous medium following Equation (4) is −49.57 kcal/mol. All these results predict that MNT and similar clay minerals can be strong candidates for trapping the 2,4-D pollutant and its residues in soils and in the whole Earth environment.

**Figure 4:**
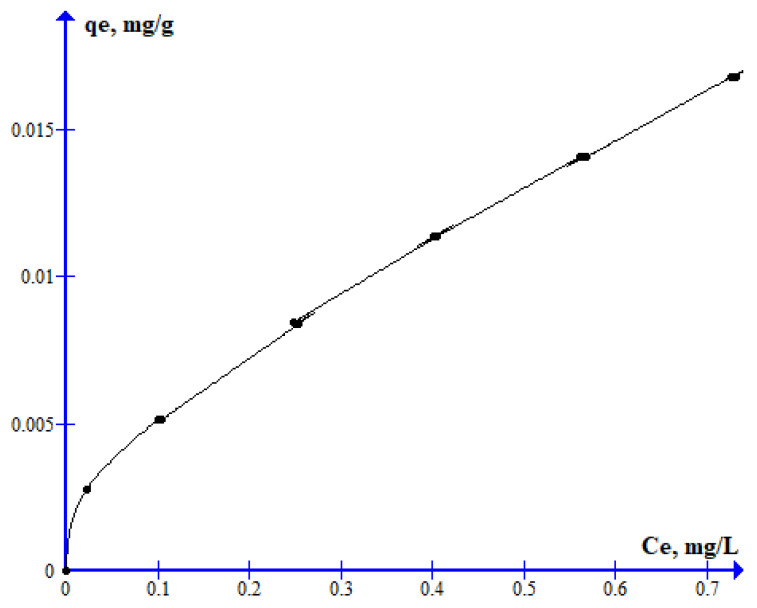
Adsorption isotherm of 2,4-D on clay-rich soil.

This process was experimentally studied by exploring the adsorption of 2,4-D on clay-rich soils. This process was performed at pH = 6.7, where the 2,4-D is as an anion. Moreover, we included the presence of Ca^2+^ in the solutions in order to reproduce a similar medium from our above intercalation calculations. The adsorption of 2,4-D increases with the initial concentration of herbicide (Figure 4). The adsorption isotherm curve is not linear, but rather type L. This indicates a physical and multilayer adsorption. With low-concentration solutions, the slope of this curve is very high, showing a high affinity of the herbicide and the adsorbent.

This isotherm was fitted to the Langmuir, Temkin, and Freundlich models for comparison purposes. The model with the best fitting is Freundlich (Table 3), indicating a multilayer adsorption. Therefore, our experimental results corroborated our theoretical calculations predicting the high adsorption capacity of clay minerals for intercalating 2,4-D.

### 2.2. Vibrational Analysis: IR Frequencies

Conformer I of the 2,4-D molecule had C_1_ symmetry, and as expected, they presented (19 × 3–6) 51 normal vibrations modes. The structure of the dimer possessed a C_s_ symmetry and (38 × 3–6) 108 normal mode vibrations, with all of them being IR active. The frequencies of the isolated molecule, dimer, and crystal structure were calculated and assigned with CASTEP and compared with the experimental data (Table 4). The main differences between theoretical and experimental frequency values come from the lack of non-bonding intermolecular interactions in the isolated molecule, whereas most of the experimental spectra come from the crystal structure. Currently, most of experimental FT-IR analysis are performed on solid samples, and many theoretical works on molecules frequencies are compared with experimental values obtained on solid samples, not in the gas phase, where the intermolecular interactions are different. However, in this work, we showed the frequencies of 2,4-D in different states (Table 4), as an isolated molecule, as a dimer, and as a solid crystal phase and compared them with the experimental values obtained in the gas phase, non-polar dissolution, and solid state in contrast to previous works.

#### 2.2.1. Region 4000–1900 cm^−1^

This region is specific to the ν(OH) and ν(CH) stretching vibration modes. The calculated frequency for the ν(OH) stretching in the 2,4-D molecule was 3636 cm^−1^, which is related with the experimental value of 2,4-D in the gas phase at around 3580 cm^−1^ [25]. However, the range of frequency decreased to 3251 when the 2,4-D was in non-polar dissolution due to various possible intermolecular interactions [19]. These interactions were confirmed with the band at 2495 cm^−1^ as the result of the dimerization process of 2,4-D [19]. Our calculations corroborated these assignments with bands at 2782–2628 cm^−1^ in the dimer and 2691–2576 cm^−1^ in the crystal structure, where hydrogen bonds were formed between the carboxylic H and O atoms. In the crystal structure, the intermolecular interactions altered the frequencies of some vibration modes. The numerous bands and the broadening of some of them in the frequency range of 3200–2500 cm^−1^ of the IR spectrum of 2,4-D [26,27,28] hint at the clustering of units in forming dimers. In other words, the formation of clusters was very likely. So, a bathochromic shift was consequently produced with respect to the isolated molecule frequencies.

In the ν(CH) mode bands, our calculations can distinguish the different H atoms of the benzenic ring. The H atom between both Cl atoms appeared at higher frequencies (3157–3140 cm^−1^) than the H atom in the meta position with respect to the alkoxy group (3138–3136 cm^−1^), probably due to the electric field of both Cl atoms. In the crystal structure, this band appeared at higher frequencies due to the additional electric field of the vicinal carbonyl groups in the crystal lattice (3157 cm^−1^). The lowest frequency of this mode corresponded to the H atom in the ortho position with respect to the alkoxy group (3120–3126 cm^−1^) (Table 4). These vibration modes showed similar frequencies in isolated molecule, dimer, and crystal states since these bonds did not participate in the intermolecular interactions.

The ν(C–H) modes of the CH_2_ groups were assigned to the bands at 3028–3019 and 2980–2955 cm^−1^ to antisymmetric and symmetric modes, respectively. In the crystal structure, these modes appeared at higher frequencies due to interactions with the O atoms of the vicinal molecules.

#### 2.2.2. Region 1800–1200 cm^−1^

In this region, the ν(C=O) mode appeared at lower frequencies in the crystal structure (1660–1611 cm^−1^) than the isolated molecule (1777 cm^−1^) due to the hydrogen bonds between this group and the OH groups of the vicinal molecules in the crystal lattice. The same was observed in the dimer (1681 cm^−1^). In this dimer formation, an eight-atom cycle was formed, and the stretching mode also implied a deformation of this cycle. This effect can be experimentally observed in this mode, which appears at 1810–1760 cm^−1^ in the gas phase, at 1739 cm^−1^ as a dimer in dissolution, and at 1733–1667 cm^−1^ in the solid state. The calculated vibrations of both groups are coupled in the crystal lattice, distinguishing the symmetrical and anti-symmetrical modes (Table 4).

In the molecule, the C=C stretching modes were at 1572–1554 cm^−1^, which were coupled to the other vibration modes, such as to the ring deformations in the plane and δ(CH) in-plane bending. No significant differences were observed between the different phases. The δ(OH) in-plane mode appeared at higher frequencies in the crystal structure than the dimer, and this one was higher than in the isolated molecule, following the sequence: 1466–1441 > 1431 > 1288–1253 cm^−1^.

### 2.3. Raman Spectrum

**Figure 5:**
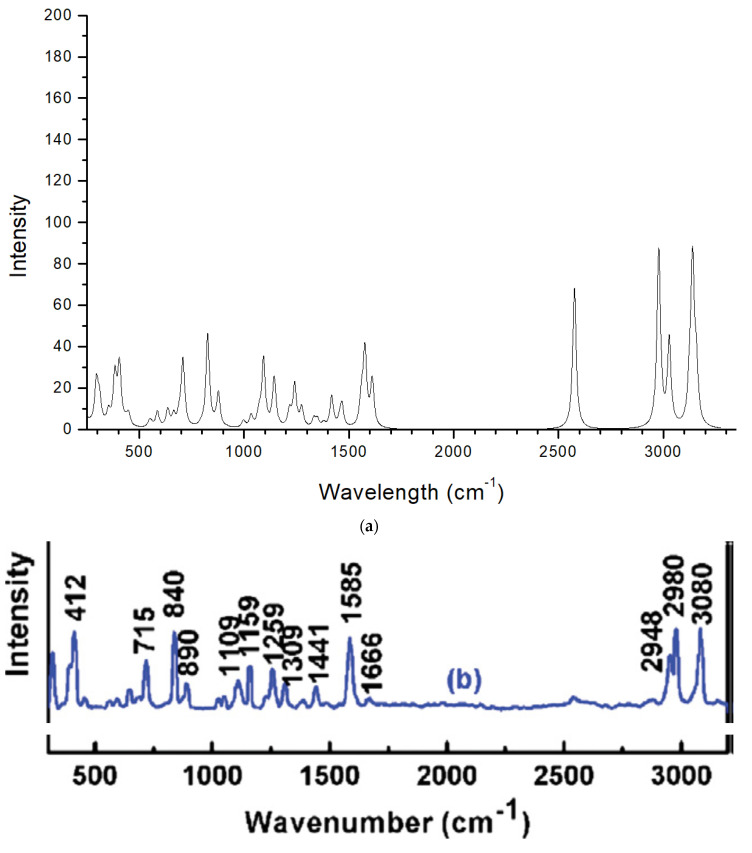
Raman spectrum calculated (CASTEP) for the optimized 2,4-D crystal structure (**a**) and experimental from Ref. [29] (**b**).

The Raman spectrum of the crystal structure of 2,4-D optimized with DFT was calculated (Figure 5). Most of the bands are consistent with the experimental spectrum [29], with the only difference being in the intensity of the band at 2570 cm^−1^, probably due to different conditions being used in the experiment and calculations and the small theoretical model not allowing disorders.

### 2.4. Experimental NMR Analysis

**Figure 6:**
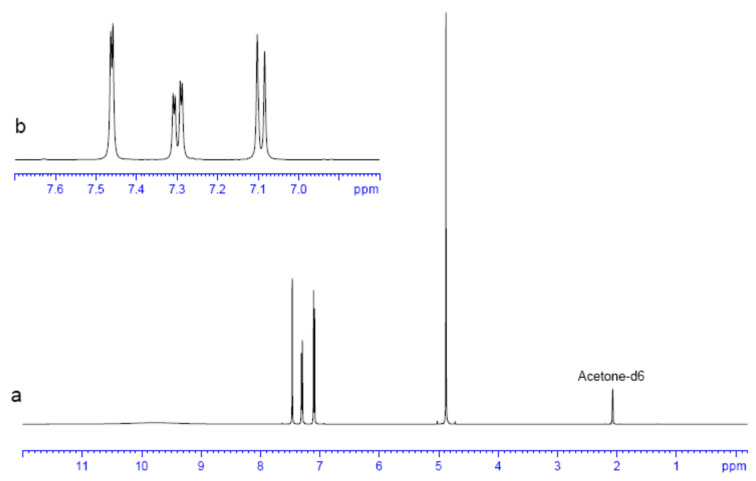
^1^H NMR spectrum of 2,4-D in solution (**a**) and from 7.0 to 7.6 ppm (**b**).

In Figure 6 and Table 5, the ^1^H chemical shifts (δ) are depicted as being consistent with previous results [21,27]. These shifts were assigned to the H atoms of 2,4-D based on multiplicity and chemical shifts. The chemical shifts from 7.09 to 7.47 ppm can be assigned to the phenyl H atoms (Figure 6b). The H(11) (numbering from Figure 1) appeared at 7.47 ppm due the deshielding effect of both Cl substituents at β position. The δ of H(13) appeared at 7.29 ppm due to the partial deshielding effect of one Cl atom. This proton showed a doublet of doublets due to a double coupling, one with the H(11) at meta position with *J* = 2.5 Hz, and other with H(10) at ortho position with higher coupling constant (*J*). The proton H(10) appeared at 7.09 ppm as a doublet with a coupling with the vicinal H atom at ortho position. The highest intensity signal at 4.87 ppm was assigned to CH_2_ (Figure 6a). This δ value could come from the deshielding from the ether O(15) atom and the carboxylic group. Finally, the broad and very low intensity chemical shift at 9.8 ppm can be assigned to the acidic H atom.

**Figure 7:**
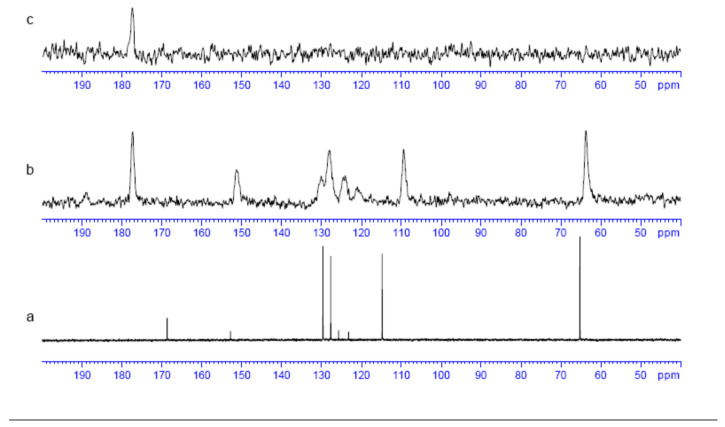
^13^C NMR spectra in solution: liquid phase (**a**); crystal phase (**b**); and high-power ^13^C decoupling (HPDEC) of the crystal phase of 2,4-D (**c**).

The ^13^C CPMAS spectra of 2,4-D in dissolution (Figure 7a) and solid state (Figure 7b,c) were recorded, and the chemical shifts are presented in Table 5. The C atoms (1), (2), (5), (7), and (8) were assigned using chemical shifts and proton–carbon correlations on the HSQC 2D map. The three quaternary carbons (C(4), C(6), and C(12)) are discriminated by long range couplings on Heteronuclear Multiple-Bond Correlation (HMBC) experiment. The carboxylic C atom was found at the largest chemical shift (168.7 ppm), and the methyl C atom at the lowest δ (65.3 ppm). All aromatic C atoms appeared in the range of 114.8–152.9 ppm, yielding C(4) with the highest δ, due to the deshielding effect of the substituent (–O-CH_2_-COOH). C(8) also appeared with a high δ because of the deshielding of the two Cl atoms in β.

In solid state, the ^13^C NMR spectrum is similar to that in dissolution with only small differences in intensities and chemical shifts. The broadening infrastructure of the signals was due to different local environments in the crystal lattice. The chemical shift of the aromatic C atom C(7) (in meta position with with respect to the Cl atoms) and the CH_2_ group in solid state was slightly lower than in dissolution due to the different local environments in each case. Similar differences were observed between the dimer in solid state and the isolated dimer where the aromatic rings have different orientations and π-π interactions between the aromatic rings in a crystal state (Figure 1 and Figure 2). The δ of the carboxylic C atom in the solid state is higher than in dissolution due to the effects of the intermolecular hydrogen bonds, indicating that they are stronger than in dissolution. Considering the similarity of spectra at both phases (Figure 7a,b), the 2,4-D molecule forms dimers in the crystal lattice. When the HPDEC spectrum was recorded, all chemical shifts disappeared, with the exception of the carboxylic C chemical shift, indicating that the relaxation time of this band strongly increased. This acceleration can be the result of a double proton transfer into the dimeric form. This led to a very rapid proton exchange between both monomers as predicted using the NMR spectra. The carbonyl group of the solvent (acetone) can likely solvate the carboxylic groups, decreasing the strength of the intermolecular H bonds of the dimer in the solution. This can explain the chemical shift difference in the carboxylic C atoms between the solid state and when in the solution.

Moreover, the high dissociation energy of the dimer form of 2,4-D would indicate the solvation could not be strong enough to destroy both hydrogen bonds in the dimer.

## 3. Methods

### 3.1. Computational Details

The molecular geometry at the gas phase was optimized starting from the geometry of the molecule extracted from the crystal structure [20]. All the calculations were performed within DFT. The isolated molecule and the dimer structure were optimized by using the hybrid functional B3LYP [30,31] and the 6-311++G(d,p) basis set [32,33,34]. For the vibrational studies of the monomer and dimer of 2,4-D, we established calculations for the geometries and harmonic frequencies in cartesian coordinates of the most stable conformers to confirm the true minimum energy critical point. The systems studied in this work are neutral molecules. The basis set superposition errors (BSSEs) in the total energies of the cyclic dimer were corrected using the counterpoise method [35]. All computations in the gas phase were carried out with the GAUSSIAN09 package [36]. The geometries were represented with the GAUSSVIEW program [37].

The crystal structure was taken from experimental crystallographic studies [20,24] (CCDC 695525). This structure was transformed to a P1 symmetry form, and the unit cell was optimized at the DFT level by using the CASTEP code with 3D periodical boundary conditions based on plane waves [38,39]. The generalized gradient approximation (GGA), Perdew–Burke–Ernzerhof (PBE) [40] correlation exchange functional, and ultrasoft pseudopotentials were used [41]; the Tkatchenko corrections for long-range dispersion interactions [42] and cutoff energies of 490 and 630 eV were also used. For the spectroscopic calculations, the structure was optimized with norm-conserving pseudopotentials with the Koelling–Harmon relativistic treatment using a cutoff energy of 990 eV and 2 × 2 × 2 *k* points of the reciprocal Brillouin space. The vibrational analysis was calculated by atomic finite displacements with harmonic approximation. The same procedure was applied to the isolated dimer and molecule embedded in 3D periodical boxes for comparison purposes. The Raman spectrum of the crystal structure was simulated for a laser light of 514.5 nm at 10 K with a smoothing Lorentzian smearing of 20 cm^−1^.

An MNT crystal structure model was chosen with a chemical composition of the unit cell as NaSi_8_(Al_3_Mg)O_20_(OH)_4_ [43]. The octahedral Mg^2+^ cations were placed considering previous studies of cation ordering in smectites [44]. For the intercalation calculations, a supercell of 2 × 2 × 1 was created with 3D periodical boundary conditions, yielding Na_4_Si_32_(Al_12_Mg_4_)O_80_(OH)_16_.

The intercalation of 2,4-D into montmorillonite was performed with Dmol^3^ code [45] based on LCAO (Linear Combination of Atomic Orbitals) with a double-ξ basis set with polarization functions and using GGA and PBE correlation exchange functional, applying periodical boundary conditions. The Tkatchenko method was also used for dispersion corrections. DFT-based semi-core pseudopotentials (DSPPs), including relativistic correction into the core [46], were also used. The frequencies of the main vibration modes were calculated from the Hessian matrix obtained from the harmonic approximation with finite atomic displacements [47]. The effect of a solvent environment (water) was simulated by using the COSMO continuum solvation model [48] with a dielectric constant of 78.54.

### 3.2. ^1^H and ^13^C NMR Spectra

The ^1^H and ^13^C NMR spectra of analytical standards of 2,4-D (Sigma-Aldrich, St. Louis, MO, USA) were performed by using a Bruker Advance III spectrometer (Billerica, MA, USA), working at 500 MHz for protons. ^1^H spectrum was recorded in the dissolution of acetone-D6, and the ^13^C spectra was recorded in acetone dissolution and solid state by using 2D-NMR Correlation Spectroscopy (COSY), Heteronuclear Multiple-Bond Correlation (HMBC), and Heteronuclear Single-Quantum Coherence Spectroscopy (HSQC). The spectra were performed at Cross-Polarization and Magic Angle Spinning (CPMAS) and high-power ^13^C decoupling (HPDEC) under magic angle spinning at a speed of 11 KHz.

### 3.3. Experimental Sorption of 2,4-D on Clay-Rich Soils

Clay-rich soil samples taken from Mercedes, an Argentinian location, was chosen for our sorption experiments. These samples were homogenized, dried, and sieved at a mesh diameter of 2 mm. 2,4-D was purchased from Sigma-Aldrich (USA).

The batch equilibrium method was used in our adsorption essays. The adsorption isotherms were performed mixing 1 g of soil with 30 mL of aqueous solutions of 2,4-D at 0.10, 0.25, 0.50, 0.75, 1.0, and 1.255 mg L^−1^. All aqueous solutions had CaCl_2_ at 0.01 M and pH = 6.72. Blank samples without soil were used simultaneously under the same conditions in order to check the loss of 2,4-D during contact time. The mixtures were stirred for 24 h at 298 K, then centrifugated for 15 min at 3000 rpm, and the supernatant was analysed by HPLC. The averaged results from the triplicate experiments were considered. The experimental data were fitted to the Freundlich, Langmuir, and Temkin models of the adsorption isotherm [49] using the least-squares method.

## 4. Conclusions

In the present work, a structural analysis of the isolated molecule and dimer of 2,4-D acid was carried out at the DFT/B3LYP level of theory, employing a 6-311++G(d,p) basis set, finding one particularly stable conformer for the isolated 2,4-D molecule and one dimer for this conformer. Moreover, the crystal structure of 2,4-D was also calculated at the DFT level, which agrees with the experimental crystal data. The theoretical vibrational spectra of the most stable conformer of the isolated molecule, dimer, and crystal structure are also calculated at different theoretical levels. Experimental and theoretical spectra were matched for exhaustive harmonic vibrational analysis, achieving a complete assignment of the bands. ^1^H and ^13^C NMR spectra were experimentally determined. For the isolated molecule, theoretical calculations predicted that non-planar conformation is predominant, which has a high dipolar moment, that is preferred in polar solvents. This molecule forms a dimer, which is present in the crystal structure, reproducing the X-ray diffraction data. From the experiments in crystals, only one conformer appeared, which agreed with our calculated conformer. The broadening of some vibrational bands of the IR spectrum strongly suggests the existence of molecular clustering in the crystal. Our calculations pointed out that the intermolecular hydrogen bonds between the carboxylic groups in crystal structure and dimer are strong. The ^13^C NMR results showed that the dimer structure exists in the solid crystal structure. The high cohesion energy of the crystal structure can justify the stability of the dimer.

Moreover, our calculations showed that the intercalation of 2,4-D into the confined interlayer space of montmorillonite was energetically favourable. This result was experimentally confirmed with the isotherm of the adsorption and desorption of 2,4-D on clay-rich soils. Hence, we predict that this clay mineral can be a good medium for trapping the 2,4-D pollutant.

## Figures and Tables

**Table 1:** The total energy [Gaussian] values (^&^E), corrected for ZPE (^#^E) and corrected for BSSE (*E) of the optimized 2,4-D molecule and cyclic dimer.

Energy (Ha)	Molecule	Dimer-I
^&^E	−1454.7351857	−2909.496049
^#^E	−1454.6069160	−2909.237675
*E		−2909.494342

**Table 2:** Cell parameters of the experimental and calculated (CASTEP) crystal structure of 2,4-D (distances in Å and angles in °).

Parameters	*a*	*b*	*c*	α	β	γ
experimental	7.12	7.84	9.0	90.7	104.6	110.5
Method A ^a^	7.31	7.99	9.03	90.2	104.6	111.6
Method B ^b^	7.19	7.86	8.95	90.6	104.0	110.7
Method C ^c^	7.20	7.87	8.97	90.6	103.9	110.7

^a^ With ultrasoft pseudopotentials and E cutoff = 490 eV. ^b^ Ultrasoft pseudopotentials and E cutoff = 630 eV. ^c^ Norm-conserving pseudopotentials and E cutoff of 990 eV.

**Table 3:** The fitting parameters of the adsorption isotherm models.

Freundlich			Langmuir			Temkin		
K_fdes_ (L g^−1^)	1/n_des_	R^2^	K_L_ (L g^−1^)	q_m_ (m g^−1^)	R^2^	K_T_ (L g^−1^)	B (mg g^−1^)	R^2^
0.019	0.526	0.988	1.856	0.028	0.946	20.02	0.006	0.954

**Table 4:** Observed and calculated wavenumbers (cm^−1^) and assignments for molecule, dimer, and crystal structures of 2,4-D.

	Experimental		Molecule ^b^	Dimer ^b^	Crystal ^b^	Assignments ^c^
Gas ^a^	dissolution	solid				
3580	3251 ^d^ (molecule)		3636			ν(OH)
3080		3075 ^e^	3141 ^f^, 3136 ^g^, 3120 ^h^	3140 ^f^, 3136 ^g^, 3121 ^h^	3157 ^f^, 3138 ^g^, 3126 ^h^	ν(CH) ring
2940		2979 ^e^	3019	3023	3028	ν(CH_2_)*_as_* OCH_2_
		2953 ^e^	2955	2961	2980	ν(CH_2_)*_s_*, OCH_2_
	2495 ^d^ (dimer)	2700–2550 ^i^		2782*_s_*, 2628*_as_*	2691*_s_*, 2576*_as_*	ν(OH)*_s_*, with H bond with C=O.
1820, 1760	1739 ^d^	1733 ^e^,1735 ^i^, 1667 ^j^	1777	1681	1660*_s_*, 1611*_as_*	ν(C=O)
1580		1595, 1580 ^e^	1571, 1554	1569, 1555	1572,1560	ν(C=C) + δ(CH) ring
		1486–1479 ^e^	1455, 1375	1373	1458, 1379	δ(CH) (ring)
				1431	1466, 1441	δ(OH)
		1480 ^i^				δ(CH_2_*)_s_* + δ(OH)
1480		1449 ^e^, 1435 ^k^	1419	1412	1420	δ(CH_2_)*_s_*
1420			1288, 1253			δ(CH) (ring) + δ(OH) + δ(CH_2_)
		1431 ^e^			1420	δ(CH_2_)_twist_ OCH_2_
1250		1393–1298 ^e^	1355*_s_*, 1224*_as_*	1339*_s_*, (1274, 1226, 1212)*_as_*	1357*_s_*, (1282, 1238, 1212)*_as_*	δ(CH_2_)_wagging_
1320		1235 ^e^, 1234 ^i^	1234, 1136, 1086, 1032	1135, 1089	1246, 1144, 1092	δ(CH) (ring)
1280		1145 ^e^	1104			δ(OH) + δ(CH_2_)
		1105 ^e^, 1089 ^k^	1068	1073	1069	ν(C-O) + δ(CH) ring + δ(OH)
			994	993	999	γ(CH_2_) OCH_2_
				1037	1028	γ(OH)
880, 760		873–796 ^e^	856, 779	908, 858, 777	926, 861, 779	γ(CH) ring
850		838 ^e^			885	ν(C-CH_2_)

^a^ Experimental data from gas phase were not assigned but extracted from the spectrum [25]. ^b^ Calculations based on DFT/PBE-plane waves. ^c^ *s*: symmetrical; *as*: asymmetrical. ^d^ Mainly a dimer in CCl_4_ dissolution [19]. ^e^ Experimental data extracted from solid state spectrum in KBr [26] and interpreted in Ref. [16]. ^f^ Aromatic CH bond in ortho to both Cl substituents. ^g^ CH bond in meta position with respect to the alkoxy substituent. ^h^ CH bond in ortho position with respect to the alkoxy group. ^i^ From Ref. [21] in solid state extracted from spectrum. ^j^ From Ref. [27] in solid state. ^k^ From Ref. [28].

**Table 5:** ^1^H and ^13^C NMR chemical shifts of 2,4-D in acetone-6D solution.

C number (Figure 1)	δ ^1^H (ppm)	δ ^13^C (ppm)
3 (C=O)		168.7, 170.5 ^b^
1 (CH_2_)	4.87, 4.83 ^a^	65.3, 68.6 ^b^
4 (O-C_arom_)		152.9, 154.2 ^b^
6 (Cl-C_arom_)		123.3, 122.2 ^b^
8 (H-C_arom_)	7.47, 7.48 ^b^	129.6, 129.2 ^b^
12 (Cl-C_arom_)		125.8, 127.9 ^b^
9 (H-C_arom_)	7.29, 7.27 ^b^	127.7, 127.1 ^b^
7 (H-C_arom_)	7.09, 6.86 ^b^	114.8, 115.6 ^b^

^a^ Experimental data [21], where the aromatic H were not identified (7.58–7.07 ppm). ^b^ From Ref. [27].

## Data Availability

The original contributions presented in this study are included in the article, and further inquiries can be directed to the corresponding authors.

## Supplementary Materials

The following supporting information can be downloaded at: https://www.mdpi.com/article/10.3390/molecules30020367/s1, Figure S1: Conformers of the dimer of 2,4-dichlorophenoxyacetic acid; Table S1: Geometrical parameters of the optimized structures for the main 2,4-D conformers and compared with experimental values; Table S2: Geometric features of the optimized structures for the main 2,4-D dimer conformers and compared with experimental values.
