# Supplementary material for: 2,4-Dichlorophenoxyacetic Acid in the Gas and Crystal Phases and Its Intercalation in Montmorillonite—An Experimental and Theoretical Study
Source: Molecules. 2025 Jan 17;30(2):367. doi: 10.3390/molecules30020367 (PMC11768003; doi:10.3390/molecules30020367)
Supplement: Supplementary file 1 [file molecules-30-00367-s001.zip › molecules-3337973-supplementary.pdf]

SUPPLEMENTARY MATERIAL

2,4-Dichlorophenoxyacetic Acid in the Gas and Crystal Phases and Its Intercalation in Montmorillonite—An Experimental and Theoretical Study

Claro Ignacio Sainz-Díaz [1,*], Nelly L. Jorge [2], Jorge M. Romero [2], André Grand [3] and Alfonso Hernández-Laguna [1,*]

1 Instituto Andaluz de Ciencias de la Tierra (IACT-CSIC), Consejo Superior de Investigaciones Científicas, Av. de las Palmeras 4, 18100 Armilla, Granada, Spain

2 Laboratorio de Investigaciones en Tecnología del Medio Ambiente, Área de Química Física, Facultad de Cien-cias Exactas y Naturales y Agrimensura, Universidad del Nordeste, Corrientes 3400, Argentina; lidianj@exa.unne.edu.ar (N.L.J.); ing.jorgemromero@gmail.com (J.M.R.)

3 Université Grenoble Alpes, Commissariat à l'Energie Atomique, Centre National de la Recherche Scientifique, Institute for Nanoscience and Cryogenics, Systèmes Moléculaires et NanoMatériaux pour l'Énergie et la Santé, F-38000 Grenoble, France; andre.grand8@wanadoo.fr

* Correspondence: ci.sainz@csic.es (C.I.S.-D.); a.h.laguna@csic.es (A.H.-L.)

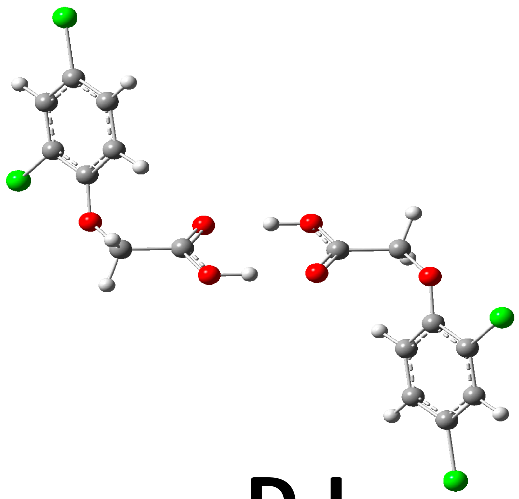

D-I

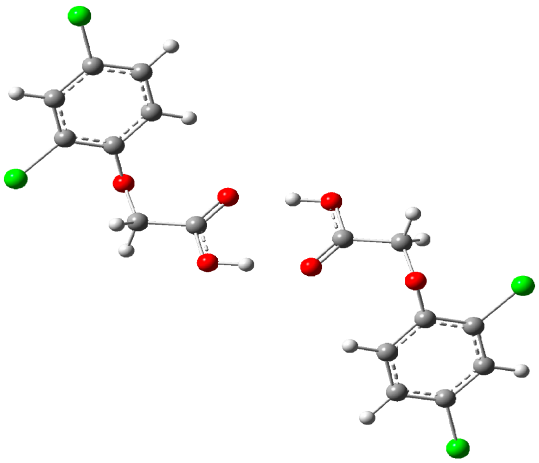

D-II

Figure S1. Conformers of the dimer of 2,4-dichlorophenoxyacetic acid.

Table S1. Geometrical parameters of the optimized structures for the main 2,4-D conformers and compared with experimental values.

Geometrical parameters	Experimental[a]	Conformer I	Conformer II
Bond lengths (Å)			
$Cl_{16}C_6$	1.741	1.748	1.753
$Cl_{14}C_{12}$	1.741	1.758	1.757
C_4C_6	1.390	1.404	1.400
C_6C_8	1.384	1.387	1.391
C_8C_{12}	1.395	1.395	1.391
$C_{12}C_9$	1.364	1.391	1.390
C_9C_7	1.413	1.395	1.390
C_7C_4	1.397	1.393	1.394
C_8H_{11}	1.030	1.081	1.080
C_9H_{13}	1.000	1.081	1.081
C_7H_{10}	1.120	1.081	1.082
C_1H_2	1.060	1.091	1.092
C_1H_{19}	1.090	1.095	1.093
$O_{17}H_{18}$	1.030	1.000	0.969
C_4O_{15}	1.365	1.365	1.374
$O_{15}C_1$	1.423	1.406	1.414
C_1C_3	1.520	1.522	1.522
C_3O_5	1.217	1.219	1.200
C_3O_{17}	1.304	1.317	1.351
Rms(Exp-i)[b]		0.028	0.032
Bond angles (°)			
$C_7C_4C_6$	119.1	119.5	118.8
$C_4C_6C_8$	122.2	120.8	121.0
$C_6C_8C_{12}$	117.3	119.3	118.8
$C_8C_{12}C_9$	122.7	121.2	121.2
$C_{12}C_9C_7$	119.1	119.3	119.1
$C_9C_7C_4$	119.6	119.6	120.9
$C_3O_{17}H_{18}$	111.5	110.6	107.9
$C_3O_5 \cdots H_{18}$	123.1	125.7	125.4
$Cl_{16}C_6C_4$	118.9	119.9	120.0
$Cl_{16}C_6C_8$	118.8	119.3	118.8
$Cl_{14}C_{12}C_8$	117.6	119.2	119.1
$Cl_{14}C_{12}C_9$	119.6	119.6	119.7
$C_6C_4O_{15}$	116.2	116.5	119.9
$C_7C_4O_{15}$	124.7	125.1	121.4
$C_4O_{15}C_1$	118.8	119.9	117.1
$O_{15}C_1C_3$	111.1	113.5	112.6
$C_1C_3O_5$	123.2	123.2	126.3
$C_1C_3O_{17}$	112.2	111.4	109.3
Rms (Exp-i)[b]		1.24	2.07
Dihedral angles (°)			
$O_{15}C_1C_3O_5$ (ϕ_1)	-3.7	0.1	2.6
$C_4O_{15}C_1C_3$ (ϕ_2)	-80.4	-80.0	-85.5
$O_5C_3O_{17}H_{18}$ (ϕ_3)	-7.1	1.0	0.0
$C_6C_4O_{15}C_1$ (Φ_4)	-179.1	-185.5	-84.0
Rms(Exp-i)[b]		5.49	47.85

[a] Ref. [20]. [b] Root mean square differences with respect to the experimental values.

Table S2. Geometric features of the optimized structures for the main 2,4-D dimer conformers and compared with experimental values.

Geometrical parameters	Experimental[a]	Structure D-I	Structure D-II
Bond length (Å)			
$Cl_{16}C_6$	1.741	1.748	1.753
$Cl_{14}C_{12}$	1.741	1.758	1.759
C_4C_6	1.389	1.404	1.400
C_6C_8	1.384	1.387	1.391
C_8C_{12}	1.396	1.391	1.389
$C_{12}C_9$	1.364	1.387	1.391
C_9C_7	1.412	1.393	1.389
C_7C_4	1.369	1.395	1.394
C_8H_{11}	1.027	1.081	1.081
C_9H_{13}	0.994	1.082	1.082
C_7H_{10}	1.118	1.082	1.082
C_1H_2	1.055	1.091	1.092
C_1H_{19}	1.088	1.095	1.094
$O_{17}H_{18}$	1.026	1.000	0.998
$O_{17}H_{18}\cdots O_5$	2.627	2.663	2.671
C_4O_{15}	1.365	1.361	1.374
$O_{15}C_1$	1.423	1.406	1.414
C_1C_3	1.514	1.522	1.522
C_3O_5	1.222	1.219	1.220
C_3O_{17}	1.303	1.317	1.317
Rms(Exp-i)[b]		0.030	0.031
**Bond angle (°)**			
$C_7C_4C_6$	119.1	119.5	118.8
$C_4C_6C_8$	122.2	120.8	121.0
$C_6C_8C_{12}$	117.3	119.3	118.9
$C_8C_{12}C_9$	122.7	121.2	121.2
$C_{12}C_9C_7$	119.1	119.3	118.8
$C_9C_7C_4$	119.6	119.6	121.1
$C_3O_{17}H_{18}$	111.9	110.0	110.6
$O_{17}H_{18}\cdots O_{36}$	173.8	179.8	178.5
$Cl_{16}C_6C_4$	118.9	119.6	120.1
$Cl_{16}C_6C_8$	118.8	119.2	118.8
$Cl_{14}C_{12}C_8$	117.6	119.3	119.0
$Cl_{14}C_{12}C_9$	119.6	119.6	119.7
$C_6C_4O_{15}$	116.2	116.5	121.4
$C_7C_4O_{15}$	124.8	125.2	119.7
$C_4O_{15}C_1$	118.8	119.9	117.2
$O_{15}C_1C_3$	111.2	113.5	113.3
$C_1C_3O_5$	123.2	123.2	123.8
$C_1C_3O_{17}$	112.7	111.4	111.1
Rms(Exp-i)[b]		2.93	3.20
Dihedral angle (°)			
$O_{15}C_1C_3O_5$ (Φ_1)	-3.7, 3.7	0.7, -0.7	2.3, -2.0
$C_4O_{15}C_1C_3$ (Φ_2)	-80.4, 80.4	-79.8, 79.8	-87.2, 86.8
$O_5C_3O_{17}H_{18}$ (Φ_3)	-7.1, 7.1	-0.7, 0.7	-0.1, 0.1
$C_6C_4O_{15}C_1$ (Φ_4)	-179.1, 179.0	-176.0, 176.0	-81.7, 81.5
Rms(Exp-i)[b]		4.2, 4.2	49.0, 49.1

[a] Ref. [20]. [b] Root mean square differences with respect to the experimental values.
